# Quantitative assessment of maternal biomarkers related to one-carbon metabolism and neural tube defects

**DOI:** 10.1038/srep08510

**Published:** 2015-03-02

**Authors:** Ke-Fu Tang, Yao-Long Li, Hong-Yan Wang

**Affiliations:** 1 The State Key Laboratory of Genetic Engineering and MOE Key Laboratory of Contemporary Anthropology, School of Life Sciences, Fudan University, Shanghai, China; 2 College of Medicine, Ningxia Normal University, Guyuan, Ningxia, China; 3 Children's Hospital Shanghai, Fudan University, Shanghai, China

## Abstract

Periconceptional supplementation with folic acid reduces the occurrence of neural tube defects (NTDs). The association between maternal abnormalities in homocysteine metabolism (e.g., hyperhomocysteinaemia, folate deficiency and low vitamin B12) and the risk of NTDs-affected pregnancies has been widely evaluated in recent years, although the results are conflicting. To investigate this inconsistency, we performed a meta-analysis of 32 studies, involving 1,890 NTD-affected mothers and 3,995 control mothers, to develop an understanding of the relationship between maternal biomarkers related to one-carbon metabolism and NTD. A random-effects model was used to calculate the ratio of means (RoM) between the cases and controls, along with the 95% confidence intervals (CIs). A significant increase in homocysteine levels was observed in NTD-affected mothers compared with controls (RoM: 1.16, 95% CI: 1.09–1.23, P = 1.8 × 10^−6^). The pooled analysis also revealed that NTD-affected mothers had significantly lower levels of folate (RoM: 0.93, 95% CI: 0.88–0.97, P = 0.002), vitamin B12 (RoM: 0.91, 95% CI: 0.87–0.95, P = 3.6 × 10^−5^) and red blood cell folate (RoM: 0.92, 95% CI: 0.86–0.98, P = 0.01). Therefore, altered plasma levels of biomarkers related to one-carbon metabolism are associated with NTD-affected pregnancies.

Neural tube defects (NTDs) are thought to be one of the most common congenital defects^1^. However, NTDs are caused by partial or complete failure of neural tube closure that causes a wide range of malformations, including anencephaly, encephalocoele and spina bifida, which further complicates the identification of the etiological factor(s)^2^. Although the underlying causes for the development and progression of NTDs have not been fully elucidated, accumulated evidence suggests that genetic variants interact with environmental factors, thereby modifying the phenotype^2^,^3^,^4^,^5^.

Numerous studies have demonstrated that periconceptional supplementation with folic acid reduces the occurrence and recurrence risk of NTDs by 50–75%^6^, although the underlying mechanisms have not been fully elucidated. However, one report has suggested that impaired homocysteine metabolism may be involved in the protective mechanism of folate^7^. Several epidemiologic studies have also reported that women with NTD-affected pregnancies have mildly elevated homocysteine levels^8^,^9^. Interestingly, homocysteine is known to be a teratogen, and can cause NTD in a chick embryo model^10^. Similarly, vitamin B12 is an active cofactor for methionine synthase, and is involved in homocysteine remethylation from the methyltetrahydrofolate donor^11^. Independent of folate, vitamin B12 deficiency has also been associated with the functional state of folate deficiency, hyperhomocysteinaemia and an increased risk of NTDs^12^,^13^,^14^.

Over the past decades, a considerable amount of research has investigated the relationship between disturbed maternal metabolism of folate and homocysteine and NTDs, hoping to identify biomarkers that could facilitate non-invasive prenatal diagnosis and prevent congenital defects. However, the maternal metabolic profile for the relevant components of the metabolic pathway are not currently known, due to the complexity of the metabolism and the lack of effective methods. Furthermore, existing studies have reported inconsistent results regarding the relationship between a disturbed maternal metabolic profile and risk of NTDs. This inconsistency may be due to insufficient power of their limited sample size, false-positive results, phenotypic heterogeneity and/or publication biases. The interpretation of these studies has also been complicated by their use of different ethnic populations, or bias in the study design. Therefore, we performed a meta-analysis of the published studies to clarify this inconsistency, and to develop an understanding of the relationship between maternal biomarkers of one-carbon metabolism and NTDs.

## Results

### Characteristics of the included studies

The search generated 1,348 publications that were available on electronic bibliographic databases (Supplementary Figure S1). Of these publications, 32 relevant studies were identified, involving 1,890 NTD-affected mothers and 3,995 control mothers; 28 assessed more than one biomarker^8^,^9^,^13^,^15^,^16^,^17^,^18^,^19^,^20^,^21^,^22^,^23^,^24^,^25^,^26^,^27^,^28^,^29^,^30^,^31^,^32^,^33^,^34^,^35^,^36^,^37^,^38^,^39^,^40^,^41^,^42^,^43^. The studies were conducted in a wide range of ethnicities, including Caucasian (42.3% of NTD-affected mothers), Asian (43.0%) and other ethnicities (14.7%). The 32 studies contained 35 datasets, including 19 retrospective comparisons and 16 prospective comparisons. Detailed characteristics of each study are in Table 1.

### Association between maternal total homocysteine levels and neural tube defects

**Figure 1:**
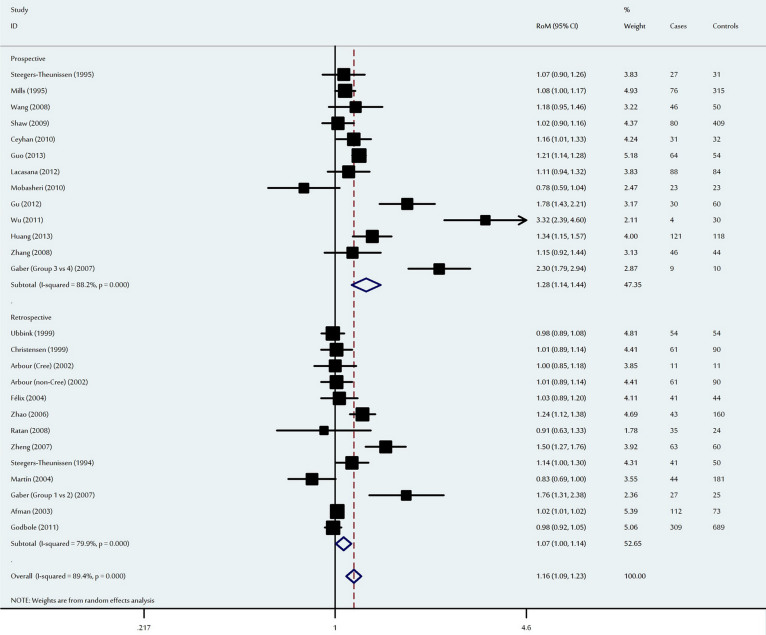
Ratio of the mean (RoM) homocysteine levels in the NTD-affected mothers compared to the control mothers and the 95% confidence intervals, as stratified by the study design.

In the primary analysis of 26 studies, NTD-affected mothers had significantly higher mean total homocysteine levels compared to control mothers (RoM: 1.16, 95% CI: 1.09–1.23, P = 1.8 × 10^−6^). Ethnic subgroup analysis revealed that the pooled RoM for Asian mothers was 1.27 (95% CI: 1.11–1.46, P = 0.001), 1.05 for Caucasian mothers (95% CI: 1.00–1.11, P = 0.07) and 1.32 for the other ethnic populations (95% CI: 1.02–1.72, P = 0.04). When analysed according to study design, a significant increase in maternal homocysteine was observed among prospective studies (RoM: 1.28, 95% CI: 1.14–1.44, P = 3.2 × 10^−5^), with a marginally significant effect among retrospective studies (RoM = 1.07, 95% CI: 1.00–1.14, P = 0.04) (Figure 1). In addition, significant heterogeneity was observed among the 26 studies in both the overall and subgroup analysis (Table 2). To evaluate the possible sources of the heterogeneity, we conducted a meta-regression analysis of ethnicity, sample size, study design and age. Ethnicity (P < 10^−4^) and study design (P < 10^−4^) were significantly correlated with the magnitude of the effect, although age (P = 0.59), number of cases (P = 0.05) and number of controls (P = 0.44) were not.

We also conducted sensitivity analysis, and the results confirmed the significant difference between maternal homocysteine levels for NTD-affected cases and controls, with RoMs and 95% CIs ranging from 1.13 (95% CI: 1.07–1.20) to 1.17 (95% CI: 1.09–1.27) (Supplementary Figure S2). Funnel plots were used to identify small study effects, and the results did not indicate the presence of publication bias among the studies (Supplementary Figure S3). The results of Egger's test (P = 0.06) confirmed the absence of publication bias.

### Association between maternal folate levels and neural tube defects

**Figure 2:**
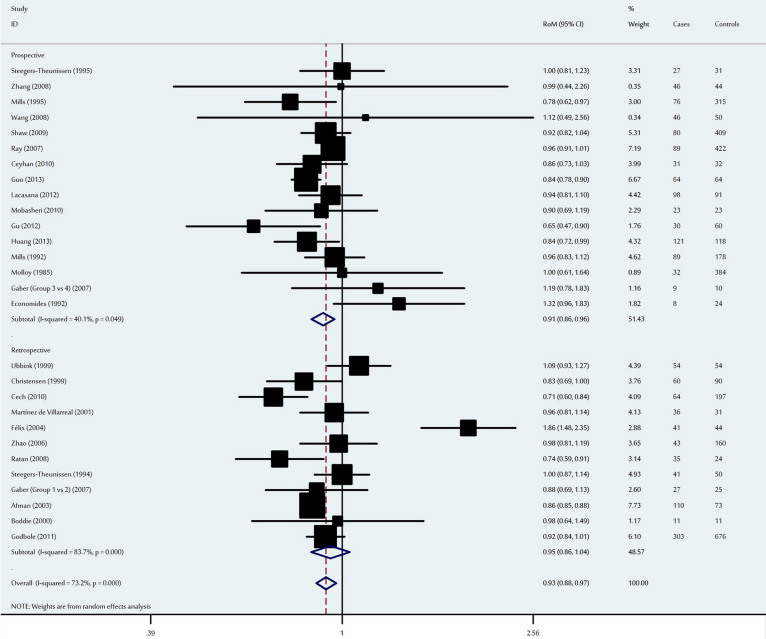
Ratio of the mean (RoM) folate levels in the NTD-affected mothers compared to the control mothers and the 95% confidence intervals, as stratified by the study design.

The meta-analysis detected a significant decrease in maternal folate levels among NTD-affected mothers, with an overall RoM of 0.93 (95% CI: 0.88–0.97, P = 0.002) (Figure 2) and statistically significant inter-study heterogeneity (P < 10^−4^). To determine the potential sources of this heterogeneity, we conducted subgroup analyses according to ethnicity and study design. When stratified by ethnicity, a significant decrease in maternal folate levels was observed among Asian and Caucasian mothers, with RoMs of 0.88 (95% CI: 0.81–0.90, P = 8.8 × 10^−10^) and 0.93 (95% CI: 0.88–0.99, P = 0.02) respectively. However, no significant difference was detected among the other ethnic populations (Table 2). When analysed according to study design, a significant decrease in folate levels was observed among prospective studies (RoM: 0.91, 95% CI: 0.86–0.96, P = 0.001), although no significant difference was detected among retrospective studies (Table 2). Ethnicity, age, number of cases, number of controls and study design were not significant sources of inter-study heterogeneity (P > 0.05 for all).

Sensitivity analysis indicated that no single study qualitatively influenced the pooled RoM, which suggests that the results of this meta-analysis were statistically robust (Supplementary Figure S4). The shape of the funnel plots were symmetrical (Supplementary Figure S5), and Egger's test did not reveal publication bias (P = 0.05).

### Association between maternal vitamin B12 levels and neural tube defects

**Figure 3:**
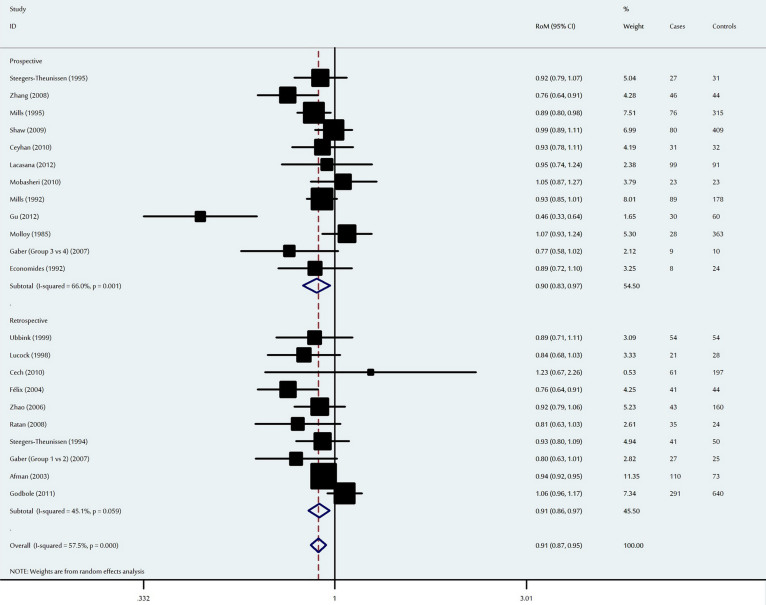
Ratio of the mean (RoM) vitamin B12 levels in the NTD-affected mothers to compared to the control mothers and the 95% confidence intervals, as stratified by the study design.

The meta-analysis detected a significant decrease in maternal vitamin B12 levels among NTD-affected mothers, with an overall RoM of 0.89 (95% CI: 0.84–0.94, P = 4.9 × 10^−5^) (Figure 3). When stratified by ethnicity, Asian mothers had a RoM of 0.79 (95% CI: 0.64–0.99, P = 0.04), Caucasian mothers had a RoM of 0.93 (95% CI: 0.90–0.98, P = 0.002), and mothers of other ethnicities had a RoM of 0.83 (95% CI: 0.75–0.92, P = 2.4 × 10^−4^). When analysed according to study design, the effects were significant for both prospective and retrospective studies, with RoMs of 0.90 (95% CI: 0.83–0.97, P = 0.008) and 0.86 (95% CI: 0.81–0.93, P = 4.5 × 10^−5^), respectively. In the meta-regression analysis, ethnicity (P = 0.07), age (P = 0.25), study design (P = 0.13), number of cases (P = 0.31) and number of controls (P = 0.13) explained the minimal level of heterogeneity.

One-way sensitivity analyses confirmed that no single study qualitatively influenced the pooled RoM (Supplementary Figure S6). The shape of the funnel plots were symmetrical (Supplementary Figure S7), and Egger's test confirmed the absence of significant publication bias (P = 0.16).

### Association between maternal red blood cell folate levels and neural tube defects

**Figure 4:**
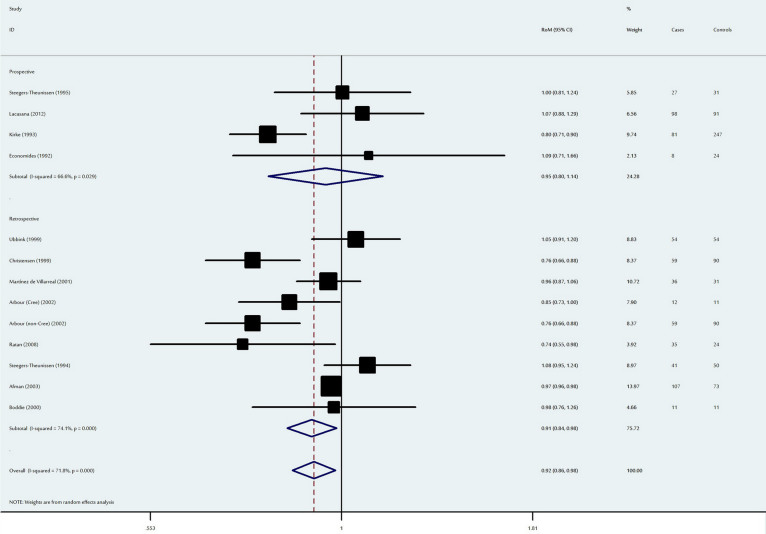
Ratio of the mean (RoM) red blood cell folate levels in the NTD-affected mothers compared to the control mothers and the 95% confidence intervals, as stratified by the study design.

Using the random effect model, a marginally significant decrease in maternal RBC folate levels was observed among NTD-affected mothers (RoM: 0.92, 95% CI: 0.86–0.98, P = 0.01) (Figure 4). When stratified by ethnicity, maternal RBC folate levels were significantly lower among Asian (P = 0.04) and Caucasian (P = 0.02) NTD-affected mothers; no significant difference was detected for the other ethnic populations (Table 2). When analysed according to study design, prospective studies had a RoM of 0.95 (95% CI: 0.80–1.14, P = 0.58), while retrospective studies had a RoM of 0.91 (95% CI: 0.84–0.98, P = 0.02).

Sensitivity analysis indicated that the results of this meta-analysis were stable, with RoMs and 95% CIs ranging from 0.903 (95% CI: 0.841–0.969, P = 0.005) to 0.934 (95% CI: 0.876–0.996, P = 0.038) (Supplementary Figure S8). No publication bias (Egger's test, P = 0.22) was observed for this overall meta-analysis (Supplementary Figure S9).

## Discussion

This meta-analysis of 32 association studies, involving 1,890 NTD-affected mothers and 3,995 control mothers, provides the first comprehensive assessment of the relationship between disturbed maternal folate and homocysteine metabolism and the risk of NTDs. Our results indicate that NTD-affected mothers have higher levels of homocysteine, and lower levels of folate, vitamin B12 and RBC folate, compared to mothers with unaffected offspring.

To identify and evaluate potential sources of heterogeneity, we performed several subgroup analyses according to ethnicity and study design. As we detected significant heterogeneity between the studies included in our meta-analysis, the results must be interpreted with caution. However, the high degree of inter-study heterogeneity may be complicated by several factors. First, heterogeneity may exist in the NTD groups, as the phenotypic severity varies with the type and level of the lesion. Second, variations in vitamin supplement content and use by the studied populations may contribute to these different results. Furthermore, the nutritional habits and dietary folic acid intake may have varied across different study populations. Third, differences in sample collection protocols (occasionally several years postpartum) may also have affected the results. The optimal period for blood sampling is in the first several weeks of pregnancy, before or soon after the failed closure of the neural tube, as nutritional changes may occur after delivery. Finally, differences in the sensitivity and/or specificity of the analytical techniques, or sample degradation during storage, may also have affected the results.

Among the prospective studies, we observed a significant increase in homocysteine metabolism in NTD-affected women. If congenital susceptibility to NTDs is partially mediated by metabolic derangement, it is possible that exposure to high levels of homocysteine may affect the closure of the neural tube at its rostral pole (i.e., during the third or fourth week after conception). Although most blood samples in the prospective studies were collected during pregnancy, they were generally collected several weeks after closure of the neural tube. Therefore, if the resulting measurement error biased our results, it likely resulted in an underestimation of the measured effects.

Several studies have previously implied that excess homocysteine, regardless of the source of its elevation, might play an independent role in the development of NTDs^9^,^29^. Other studies have reported that homocysteine overload could affect the embryo's development by interfering with the embryonic cell cycle or inducing apoptosis, thereby leading to NTDs^44^. Recently, Rosenquist et al. have reported that homocysteine increased the number of NTDs when administered to chicken embryos *in ovo*^45^. However, another study reported that homocysteine failed to induce NTDs in a standard mouse model^46^. Therefore, it has been suggested that homocysteine may indirectly affect the development of NTDs^47^, possibly through a mechanism whereby homocysteinylation damages proteins and alters their function^48^.

When we stratified our analysis by study design, significantly lower levels of folate and vitamin B12 were observed in NTD-affected mothers (vs. controls) from the prospective studies. Maternal folic acid intake substantially reduces the probability of occurrence or recurrence of NTDs^2^,^6^, and our results support this benefit. In addition, folate responsiveness is important in the development of NTDs^49^, and we observed decreased levels of RBC folate in NTD-affected mothers. RBC folate reflects the level of intracellular folate and folate turnover during the previous 4 months, and is generally considered a more useful indicator of folate status than serum folate. Similar to our results, Daly et al. have suggested that decreased maternal RBC folate in early pregnancy is a marker of NTD risk^50^, in a concentration-dependent manner. Interestingly, vitamin B12 is an important coenzyme in the metabolic pathway of homocysteine, and affects homocysteine levels by maintaining the activity of folate. Therefore, either deficiency in folate or vitamin B12 can result in elevated plasma levels of homocysteine. As vitamin B12 is mainly absorbed from dietary intake, it is possible that mothers suffer from vitamin B12 deficiency due to changes in dietary patterns during pregnancy^43^, thus further increasing the risk of NTDs. As abnormality in homocysteine metabolism is present in NTD-affected women, our findings suggest that periconceptional supplements of vitamin B12 and folic acid may be more effective in NTD prevention.

As a retrospective study, the current meta-analysis is subject to the methodological deficiencies of the included studies, and several specific details merit consideration. First, heterogeneity is a potential factor must be considered when interpreting our results. Although we failed to identify the main sources of the heterogeneity in effect size, a meta-analysis of reported data has little capacity to do so. Ideally, we would prefer to pool individual-level data to allow for efficient assessment of the sources of heterogeneity, although this would be impractical for the present study. Second, populations from geographically distinct countries were pooled according to ethnicity in the subgroup meta-analyses, which may have generated a fluctuation effect. Third, our results were based on unadjusted estimates. If each individual's raw data were available, a more precise analysis could be conducted, which could be adjusted for other co-variants, such as age, obesity, folate supplementation and other lifestyle factors.

Despite these limitations, the findings of the present study indicate that altered plasma levels of biomarkers related to one-carbon metabolism are associated with NTD-affected pregnancies. To confirm these findings, future studies should involve a prospective design, strict selection of cases and larger studies of diverse ethnic populations.

## Methods

### Identification and screening of relevant studies

Given the limited evidence available regarding other metabolism biomarkers, the present meta-analysis was restricted to studies that evaluated maternal total homocysteine, folate, vitamin B12 and red blood cell (RBC) folate. We used computer-based searches to identify epidemiologic association studies published before September 2014, which investigating at least one of the four metabolism biomarkers in NTD-affected pregnancies. The databases we searched included PubMed, ISI Web of Science, EMBASE, EBSCO, the Cochrane Library databases, and the Chinese National Knowledge Infrastructure. No language restrictions were imposed on our search.

The search included keywords relating to NTDs (e.g., “neural tube defects”, “anencephaly”, “encephalocele” and “spina bifida”) in combination with words related to the maternal biomarkers (e.g., “homocysteine”, “hyperhomocysteinemia”, “folic acid”, “folate”, “vitamin B12” and “red blood cell folate”). The titles and abstracts from retrieved articles were screened to determine their relevance, and irrelevant studies were excluded without further evaluation. For the remaining articles, the full texts were evaluated to determine whether they contained data of interest. The reference lists from all relevant publications were also hand-searched for additional eligible reports.

For inclusion, studies were required to meet all of the following criteria: (1) investigated maternal one-carbon metabolism and the risk of NTDs (2) original human studies with independent data; (3) followed a case-control or cohort study design; (4) maternal biomarkers were measured separately for cases and controls using a reliable assay; (5) described the assessment methods, equipment and protocols, or provided reference to them; (6) the results were expressed as, or could be estimated into, mean and standard deviation (SD). Major exclusion criteria were: (1) case-only studies and (2) overlapping data. Case reports, editorials, and review articles were also excluded. If more than one paper was published studying the same sample series, only the study with the largest sample size and the most detailed information was included. Studies with different ethnic groups were considered as individual studies for our analyses.

NTD-affected pregnancies were defined as women whose children had NTDs, including live births, stillbirths, and prenatally diagnosed foetuses. Control subjects were defined as healthy women with unaffected pregnancies, or those who had a normal child-bearing history without any history of congenital malformation.

### Data extraction

The two authors independently extracted the following information from each study according to a fixed protocol: first author, year of publication, ethnicity of the study population, clinical characteristics, matching criteria, study design, quantified method of biochemical analyses, diagnostic criteria for NTDs, number of cases and controls, collection strategy for maternal biological specimens and the mean and SD values for metabolites from the cases and controls. When only median and range were reported in the text, a conversion formula^51^ was used to calculate the mean and SD. Data reports from the two reviewers were than compared to identify any inconsistency, and differences were resolved by further discussion among all authors through consensus.

### Statistical analysis

As metabolite concentrations were measured using different methods and reported in various units across different studies, we therefore used a ratio method to meta-analyse continuous values for each metabolite^52^,^53^,^54^. In brief, the metabolite concentrations difference in the means between NTD cases and controls for each study was expressed as the ratio of the mean (RoM). RoM was defined as the mean value of the NTD group divided by that of the control group. The variance of RoM was estimated using the delta method^55^, and the values were pooled using inverse-variance weighting. Heterogeneity across studies was assessed using Cochran's chi-square Q test and the I^2^ test^56^. Random effects models were used to calculate the overall results, as these assume a genuine diversity in the results of various studies, and typically provide wider confidence intervals (CIs) when significant inter-study heterogeneity exists^57^. The 95% CIs were constructed using Woolf's method, and the significance of the overall RoM was determined using the Z-test.

Sources of heterogeneity were investigated by stratified meta-analyses based on ethnicity and study design (prospective vs. retrospective study). A prospective study was defined as a study where the maternal blood sample was collected before delivery, and a retrospective study was defined as a study where the maternal blood sample was collected postpartum. Ethnic groups were defined as Asian (i.e., Chinese, Japanese, and Indian), Caucasian (i.e., subjects of European ancestry) or other ethnic populations. In addition, ethnicity, sample size and study design were analysed as covariates in a meta-regression. To assess the stability of the results, sensitivity analysis was performed by removing each individual study in turn from the total, and re-analysing the remaining studies. Funnel plots and Egger's linear regression test were used to evaluate potential publication bias^58^. The type I error rate was set at 0.05 for two-sided analysis. All the analyses were performed using Stata 10.0 software (STATA Corp., College Station, TX, USA).

## Author Contributions

Conceived and designed the study: K.F.T.; Performed the experiments: K.F.T., Y.L.L. and H.Y.W.; Contributed material/analysis tools: K.F.T. and Y.L.L.; Statistical analyses and paper writing, revising: K.F.T. and H.Y.W.

## Supplementary Material

Supplementary Information Supplementary information

## Figures and Tables

**Table 1:** Characteristics of studies included in meta-analysis

First author	Year	Ethnic population	Biomarkers	Study design	No. of cases/controls	NTDs-affected mothers		Control mothers		
						Age	Gestational age (week)	Age	Gestational age (week)	Matching
Steegers-Theunissen	1995	Dutch	tHcy, VB12, folate, RBC folate	Prospective	27/31	30.5	22.3	37.8	15.9	Location
Ubbink	1999	African	tHcy, VB12, folate, RBC folate	Retrospective	54/54	26.5	nr	28.3	nr	Location, socioeconomic
Groenen	2004	Dutch	tHcy, VB12, folate, RBC folate	Retrospective	45/83	32.3	nr	33.3	nr	Location
Zhang	2008	Chinese	tHcy, VB12, folate	Prospective	46/44	nr	nr	nr	nr	Location
Mills	1995	Irish	tHcy, VB12, folate, RBC folate	Prospective	76/315	nr	nr	nr	nr	Location
Lucock	1998	British	tHcy, VB12, folate, RBC folate	Retrospective	21/28	nr	nr	nr	nr	Location, age, social class
Martín	2004	Spanish	tHcy	Retrospective	44/181	36.0	nr	39.0	nr	Location
Martínez de Villarreal	2001	Mexican	folate, RBC folate	Retrospective	36/31	33.6	nr	26.9	nr	Ages, neighbourhoods
Wang	2008	Chinese	tHcy, folate	Prospective	46/50	nr	nr	nr	nr	Location
Christensen	1999	Canadian	tHcy, folate, RBC folate	Retrospective	61/90	nr	nr	nr	nr	Location
Shaw	2009	American	tHcy, VB12, folate	Prospective	80/409	25–29	15–18	30–34	>20	Location, ethnicity
Arbour	2002	Norwegian	tHcy, RBC folate	Retrospective	73/101	nr	nr	nr	nr	Location, age, ethnicity
Félix	2004	Brazilian	tHcy, VB12, folate	Retrospective	41/44	25.8	nr	25.6	nr	Location
Zhao	2006	American	tHcy, VB12, folate	Retrospective	43/160	nr	nr	nr	nr	Location, ethnicity
Ceyhan	2010	Turkish	tHcy, VB12, folate	Prospective	31/32	26.6	nr	28.3	nr	Location, gestation
Guo	2013	Chinese	tHcy, folate	Prospective	64/64	nr	nr	nr	nr	Location, age, gestation
Ratan	2008	Indian	tHcy, VB12, folate, RBC folate	Retrospective	35/24	nr	nr	nr	nr	Location, socio-economic and nutritional status
Lacasaña	2012	Mexican	tHcy, VB12, folate, RBC folate	Prospective	99/91	nr	nr	nr	nr	Location, maternity clinic, date of birth
Mobasheri	2010	Iran	tHcy, VB12, folate	Prospective	23/23	24.0	nr	26.5	nr	Location
Gu	2012	Chinese	tHcy, VB12, folate	Prospective	30/60	27.6	26.7	28.7	26.4	Location
Huang	2013	Chinese	tHcy, folate	Prospective	121/118	nr	nr	nr	nr	Location
Zheng	2007	Chinese	tHcy	Retrospective	63/66	nr	nr	nr	nr	Location
Wu	2011	Chinese	tHcy	Prospective	4/30	nr	24–32	nr	24–40	Location
Steegers-Theunissen	1994	Dutch	tHcy, VB12, folate, RBC folate	Retrospective	41/50	35.4	nr	33.3	nr	Location
Molloy	1985	Irish	VB12, folate	Prospective	32/384	nr	nr	nr	nr	Location
Gaber	2007	Egyptian	tHcy, VB12, folate	Prospective & Retrospective	36/35	27.0	nr	nr	nr	Location
Mills	1992	Finnish	VB12, folate	Prospective	89/178	nr	nr	nr	nr	Location
Economides	1992	British	VB12, folate, RBC folate	Prospective	8/24	nr	17.9	nr	18.9	Location
Afman	2003	Dutch	tHcy, VB12, folate, RBC folate	Retrospective	112/73	42.9	nr	35.6	nr	Location
Boddie	2000	American	folate, RBC folate	Retrospective	11/11	26.5	nr	27.2	nr	Location
Godbole	2011	Indian	tHcy, VB12, folate	Retrospective	309/689	24	23.0	25	37.3	Location
Ray	2007	Canadian	folate	Prospective	89/422	28.6	nr	29.8	nr	Location

tHcy: total homocysteine; VB12: vitamin B12; RBC: red blood cell; nr: not reported.

**Table 2:** Meta-analysis of maternal biomarkers related to the effect of one-carbon metabolism on risk of neural tube defects

Overall and subgroups analyses	tHcy					Folate					Vitamin B12					RBC folate				
	No. of cases/controls	RoM (95% CI)	P(Z)	P(Q)	I^2^ (%)	No. of cases/controls	RoM (95% CI)	P(Z)	P(Q)	I^2^ (%)	No. of cases/controls	RoM (95% CI)	P(Z)	P(Q)	I^2^ (%)	No. of cases/controls	RoM (95% CI)	P(Z)	P(Q)	I^2^ (%)
All	1,547/2,811	1.16 (1.09–1.23)	1.8 × 10^−6^	<10^−5^	89	1,694/3,690	0.93 (0.88–0.97)	0.002	<10^−4^	73	1,270/2,875	0.91 (0.87–0.95)	3.6 × 10^−5^	<10^−3^	57	628/827	0.92 (0.86–0.98)	0.01	<10^−5^	72
Ethnicity																				
Asian	772/1,184	1.27 (1.11–1.46)	0.001	<10^−5^	90	699/1,091	0.88 (0.81–0.90)	8.8 × 10^−10^	0.41	2	456/823	0.84 (0.70–1.02)	0.07	<10^−4^	84	35/24	0.74 (0.55–0.98)	0.04	NA	NA
Caucasian	556/1,410	1.05 (1.00–1.11)	0.07	0.003	64	666/2,147	0.93 (0.88–0.99)	0.02	0.001	67	523/1,631	0.94 (0.92–0.95)	<10^−10^	0.60	0	405/627	0.90 (0.82–0.98)	0.02	<10^−4^	78
Others	219/217	1.32 (1.02–1.72)	0.04	<10^−4^	92	329/452	1.04 (0.84–1.28)	0.74	<10^−5^	87	291/421	0.83 (0.75–0.92)	2.4 × 10^−4^	0.50	0	188/176	1.00 (0.93–1.08)	0.99	0.47	0
Study design																				
Prospective	645/1,300	1.28 (1.14–1.44)	3.2 × 10^−5^	<10^−5^	88	869/2,255	0.91 (0.86–0.96)	0.001	0.05	40	546/1,580	0.90 (0.83–0.97)	0.008	0.001	66	214/393	0.95 (0.80–1.14)	0.58	0.03	67
Retrospective	902/1,511	1.07 (1.00–1.14)	0.04	<10^−5^	80	825/1,435	0.95 (0.86–1.04)	0.24	<10^−5^	84	724/1,295	0.91 (0.86–0.97)	0.004	0.06	45	414/434	0.91 (0.84–0.98)	0.02	<10^−5^	74

P(Z): Z test used to determine the significance of the overall OR.

P(Q): Cochran's chi-square Q statistic test used to assess the heterogeneity in subgroups.

NA: not available; RBC: red blood cell; RoM: ratio of the mean; tHcy: total homocysteine.
