# Supplementary material for: Quantitative assessment of maternal biomarkers related to one-carbon metabolism and neural tube defects
Source: Sci Rep. 2015 Mar 2;5:8510. doi: 10.1038/srep08510 (PMC4345334; doi:10.1038/srep08510)
Supplement: Supplementary Information [file srep08510-s1.pdf]

Quantitative Assessment of Maternal Biomarkers related to One-Carbon Metabolism and Neural Tube Defects

Ke-Fu Tang, Yao-Long Li, Hong-Yan Wang *

*Corresponding author

Supplementary Figure S1

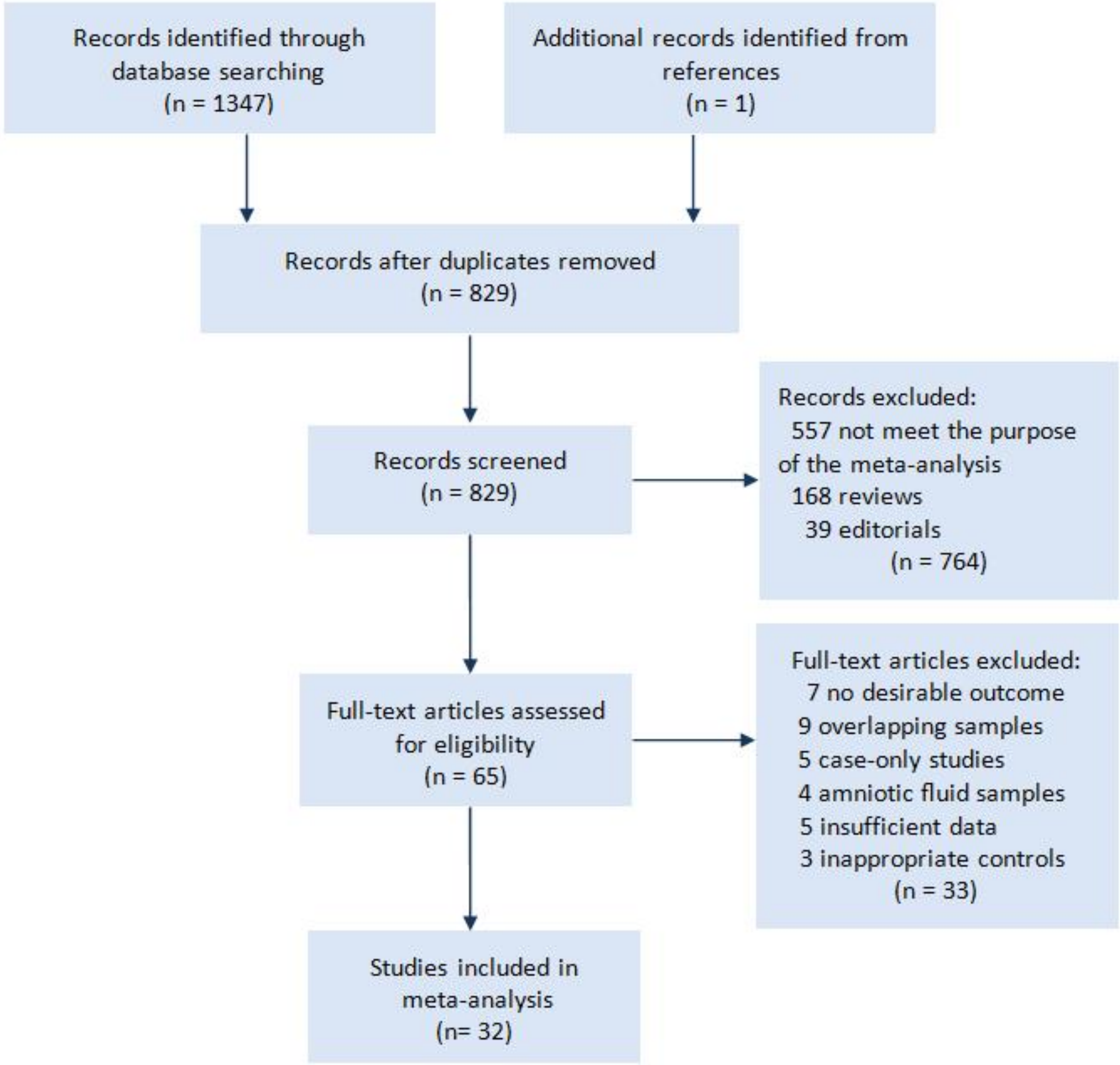

Supplementary Figure S1. Flow chart of literature search for studies examining Maternal Biomarkers related to One-Carbon Metabolism and Neural Tube Defects

Supplementary Figure S2

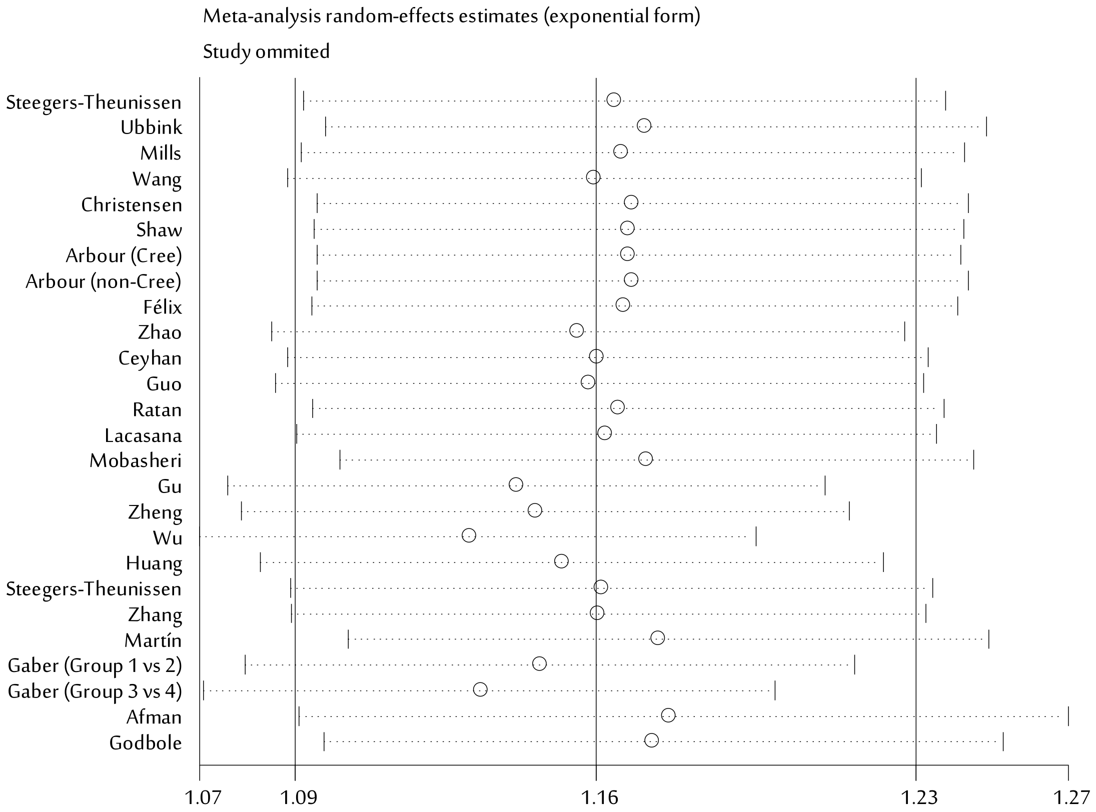

Supplementary Figure S2. Sensitivity analysis on the RoM of maternal homocysteine level in the NTDs-affected mothers compared with control mothers

Supplementary Figure S3

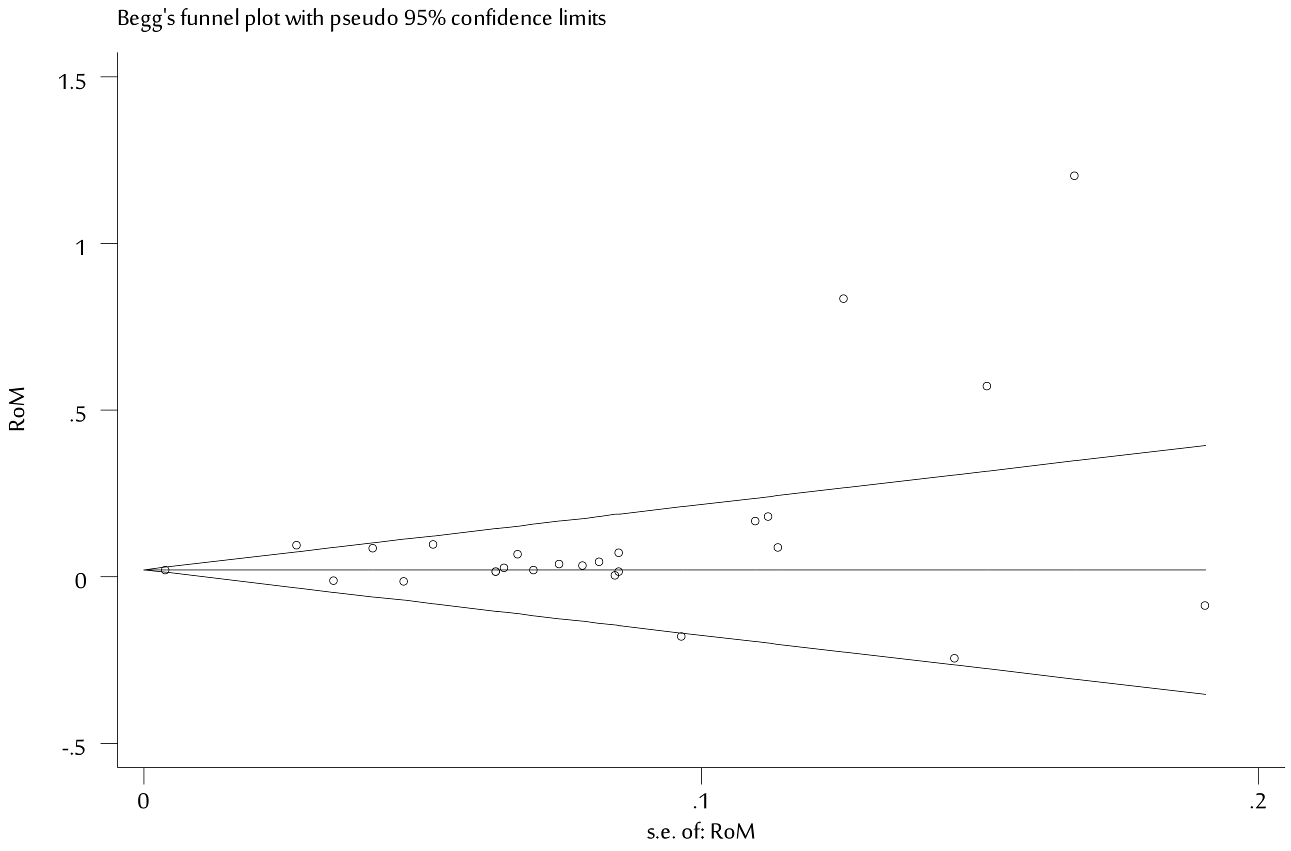

Supplementary Figure S3. Publication bias in studies of maternal homocysteine level in the NTDs-affected mothers compared with control mothers by Funnel plot.

Supplementary Figure S4

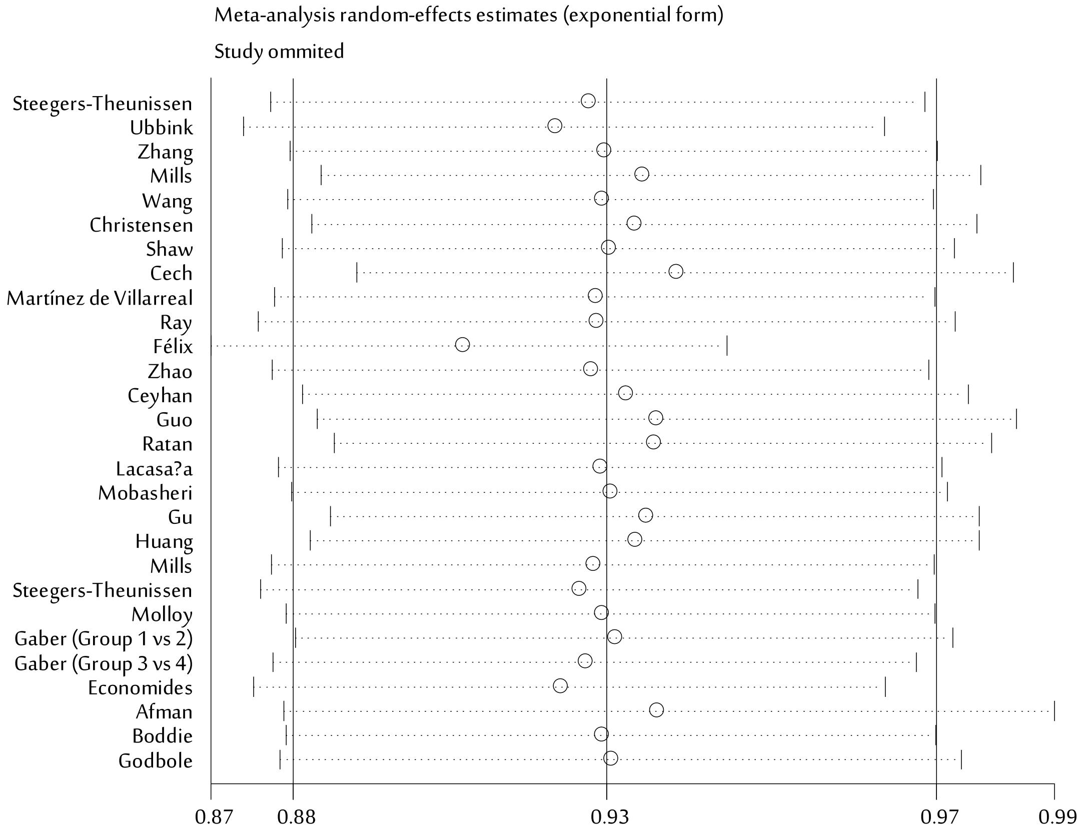

Supplementary Figure S4. Sensitivity analysis on the RoM of maternal folate level in the NTDs-affected mothers compared with control mothers

Supplementary Figure S5

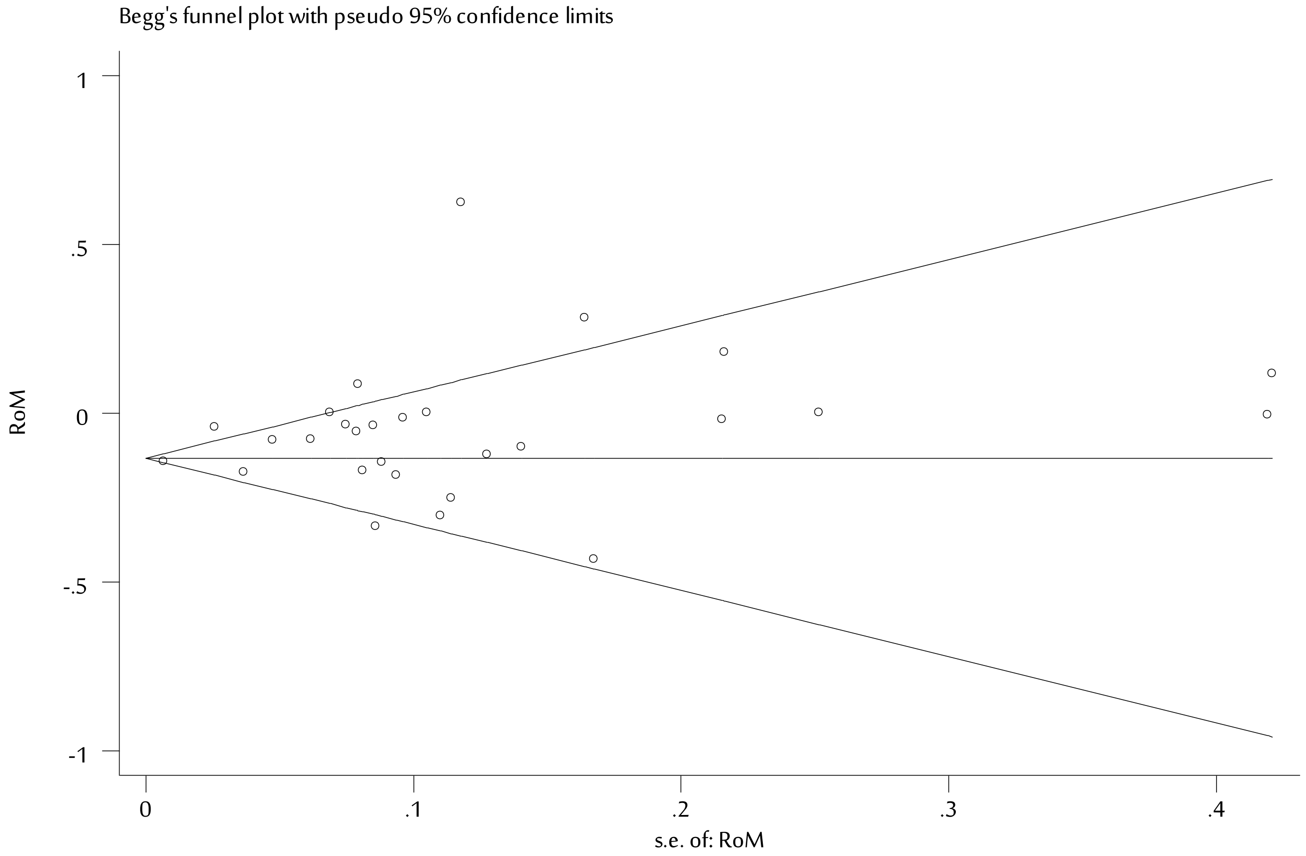

Supplementary Figure S5. Publication bias in studies of maternal folate level in the NTDs-affected mothers compared with control mothers by Funnel plot.

Supplementary Figure S6

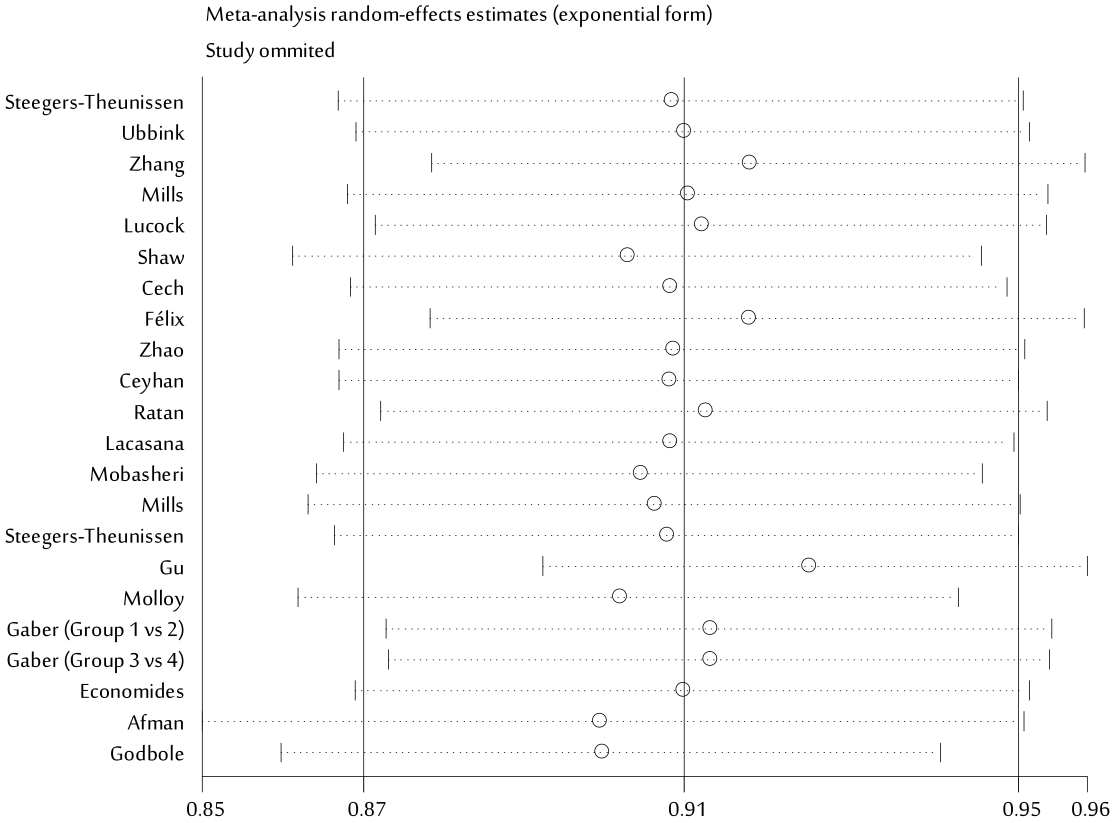

Supplementary Figure S6. Sensitivity analysis on the RoM of maternal vitamin B12 level in the NTDs-affected mothers compared with control mothers

Supplementary Figure S7

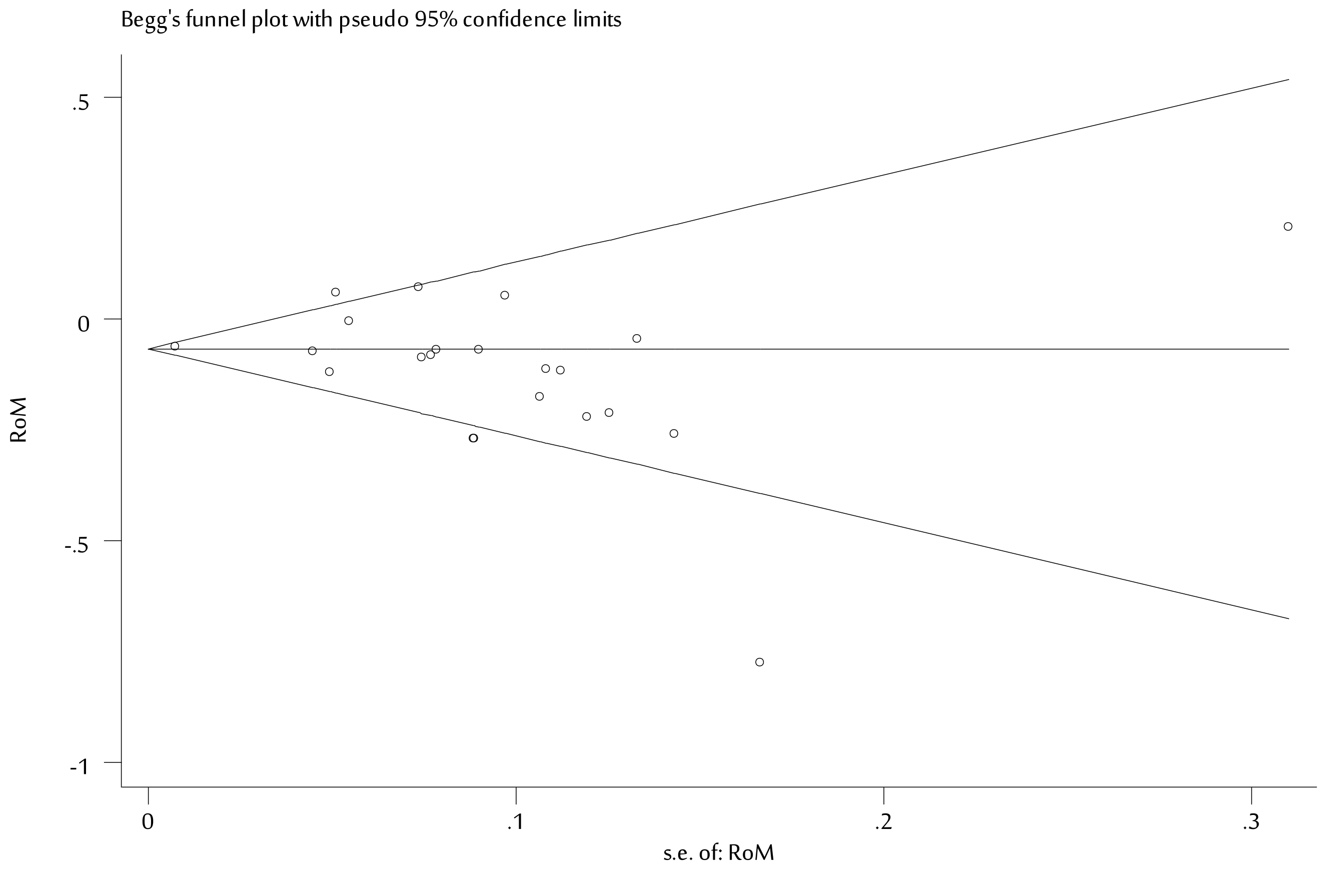

Supplementary Figure S7. Publication bias in studies of maternal vitamin B12 level in the NTDs-affected mothers compared with control mothers by Funnel plot.

Supplementary Figure S8

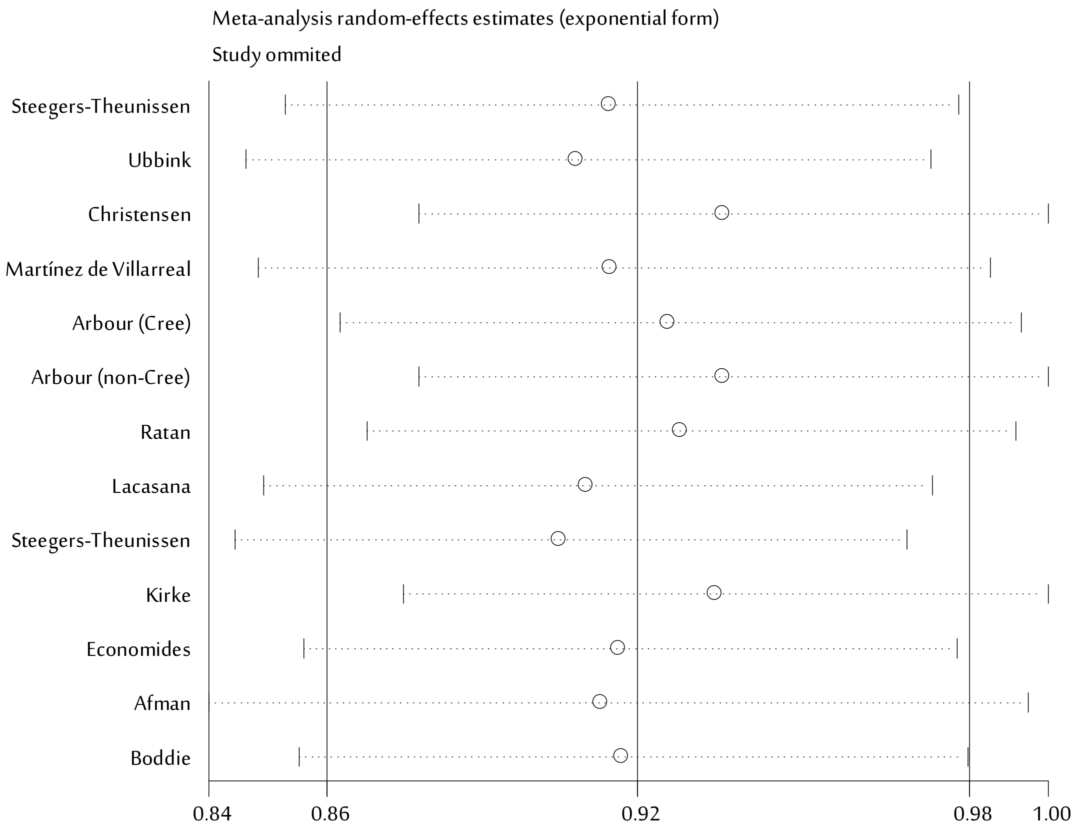

Supplementary Figure S8. Sensitivity analysis on the RoM of maternal red blood cell folate level in the NTDs-affected mothers compared with control mothers

Supplementary Figure S9

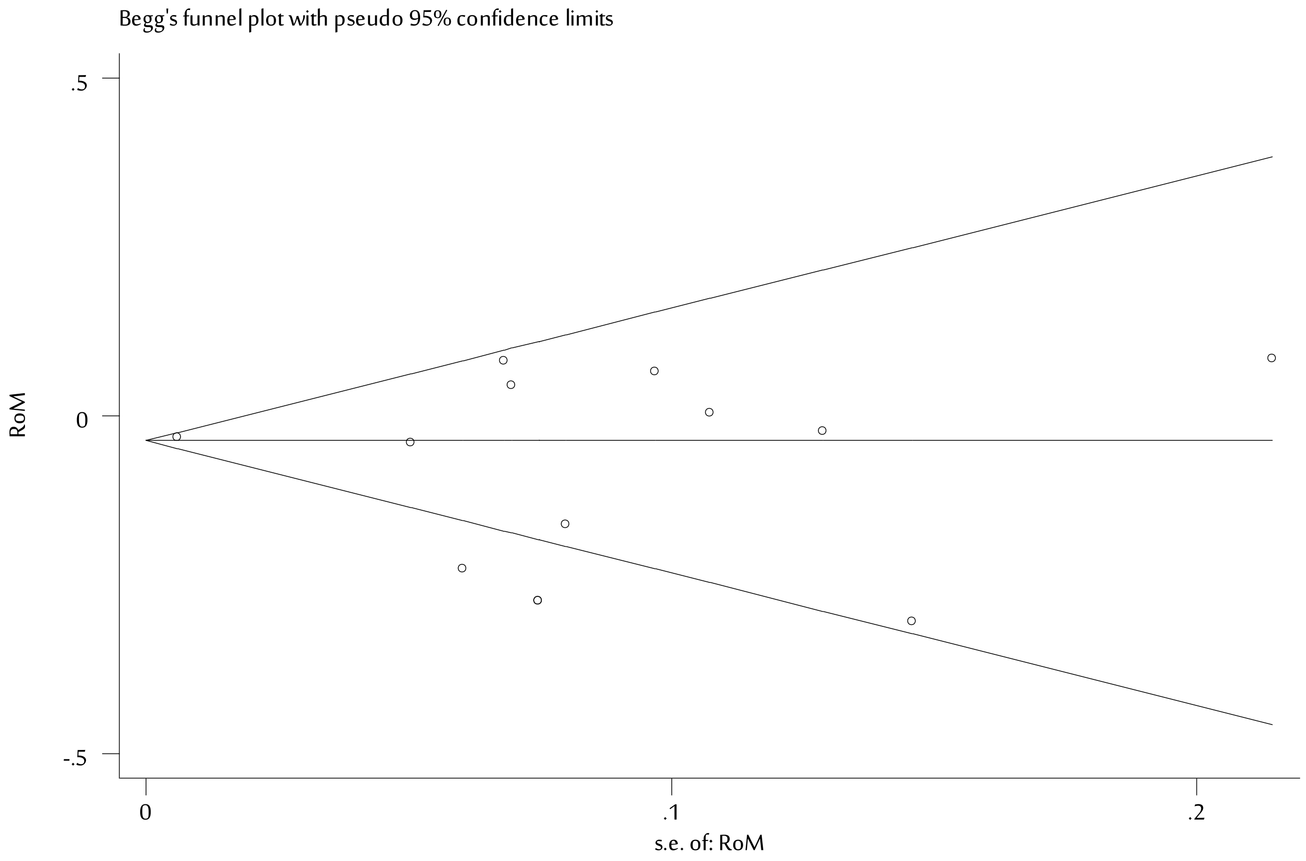

Supplementary Figure S9. Publication bias in studies of maternal red blood cell folate level in the NTDs-affected mothers compared with control mothers by Funnel plot.
